# Visitor satisfaction and development effectiveness in red tourism: evidence from Guangzhou

**DOI:** 10.3389/fpsyg.2025.1616713

**Published:** 2025-10-30

**Authors:** Kun Li, Wen-Bing Mei, Yi-Zhe Huang

**Affiliations:** ^1^School of Fine Arts and Design, Guangzhou University, Guangzhou, Guangdong, China; ^2^School of Art and Design, Guangdong Industry Polytechnic University, Guangzhou, Guangdong, China; ^3^School of Education, City University of Macau, Macau, Macao SAR, China

**Keywords:** red tourism in Guangzhou, scenic area development, effectiveness, visitor satisfaction, determinants

## Abstract

Promoting red historical culture, fostering patriotic sentiment, and strengthening cultural confidence are central tasks in China’s cultural development in the new era. As both a cradle and a vanguard of China’s modern democratic revolution, Guangzhou is endowed with abundant red historical and cultural resources. Assessing the effectiveness of red tourism site development in Guangzhou thus carries significant practical implications. Drawing on expert interviews, questionnaire surveys, and big-data statistical analysis, and using the importance–performance analysis (IPA) quadrant method, this study reaches the following conclusions: (1) The development effectiveness of Guangzhou’s red tourism sites constitutes a multidimensional composite structure, comprising three critical dimensions—red cultural connotations, management behavioral norms, and landscape and facilities—encompassing 27 specific indicators; (2) the three dimensions exert differential effects on visitor satisfaction, with red cultural connotations (*β* = 0.455) emerging as the most influential, followed by management behavioral norms (0.209) and landscape and facilities (0.114); (3) while visitors generally hold high expectations regarding the development of Guangzhou’s red tourism sites, their actual perceptions fall short, revealing a significant negative “expectation–perception” gap. Based on these findings, we propose targeted strategies to enhance site development effectiveness and visitor satisfaction, offering practical and theoretical insights for improving red tourism site development.

## 1 Research background and objectives

Red tourism is a distinct Chinese socio-systemic phenomenon encompassing economic, political, and cultural dimensions. It is a form of tourism that has developed alongside the rise of China’s tourism industry, with red culture as its defining theme (Liu, 2012; Zuo, 2014). Currently, the definition of “red culture” differs between official government sources and academic scholars (Tao and Hu, 2012). According to the National Red Tourism Development Plan Outline (2011–2015), red historical culture is defined as the advanced culture formed since 1840 through the Chinese people’s struggle against oppression, pursuit of national independence, and efforts to build a modern socialist state. It is the sum of various material and spiritual carriers that can meet the public’s needs (China, 2011).

Comparable forms of research abroad are generally categorized as red tourism, wartime tourism, and dark tourism (Carbone, 2022; Prayag et al., 2021). Scholarly studies on red tourism focuses primarily on the Chinese practice (Tao and Hu, 2012), addressing themes such as heritage preservation, communication, identity, and political education (Fokin and Elts, 2019). Red tourism itself is imbued with profound educational value: it serves as a principal means of recalling arduous revolutionary struggles, promoting the advanced revolutionary spirit, and transmitting the core values of socialism (Zhang et al., 2022). With socialism with Chinese characteristics entering a new era, advancing red historical culture and strengthening patriotic sentiment and historical-cultural confidence have become integral to national cultural development (Xu, 2016). As focal points for red cultural heritage, red tourism sites serve both as carriers of revolutionary spirit and culture and as primary arenas for cultivating and transmitting the “red gene” (Li and Zhang, 2023).

Guangzhou, celebrated as a heroic city rich in red historical culture and revolutionary tradition (Song, 2010), has long attached great importance to cultural construction. The Guangdong Provincial Party Committee, in its Four Innovations and Brilliance action plan, explicitly called for Guangzhou to be developed as a “demonstration zone for inheriting and promoting red culture.” Similarly, the Guangzhou Municipal Government has included the creation of a “red historical and cultural brand” within the tasks of its 14th Five-Year Plan. In recent years, Guangzhou has increasingly prioritized the development of red historical and cultural resources, achieving notable success in government-led restoration and preservation of historical sites. Nonetheless, challenges remain in the city’s red tourism development, including insufficient dissemination of red culture, homogenization of resource development, weak regional distinctiveness, limited product innovation, and modest regional economic spillover effects (Guangzhou Municipal Government, 2021; Liang et al., 2023).

Enhancing the effectiveness of tourism site development ultimately depends on improving visitor satisfaction. Only through high levels of perceived satisfaction can investments in infrastructure and the intrinsic value of cultural resources be translated into genuine educational, cultural, and social benefits (Vargo and Lusch, 2014). Visitor satisfaction thus constitutes a pivotal indicator of development effectiveness. Enhancing satisfaction not only strengthens the communicative and educational functions of red culture but also fosters positive word-of-mouth, attracting more visitors and promoting a virtuous cycle of sustainable development (Hou et al., 2022). Accordingly, examining the development effectiveness of Guangzhou’s red tourism sites and the factors shaping visitor satisfaction carries significant practical value. On the one hand, it enables the optimization of resource allocation and service quality, thereby enriching both the educational role and the visitor experience. On the other hand, it provides empirical support for revitalizing red cultural resources, promoting high-quality integration of culture and tourism, and strengthening both cultural confidence and social benefits (Liao et al., 2009).

As a special form of heritage tourism, the development quality of red tourism concerns not only economic benefits but also the inheritance and promotion of the mainstream national values (Liu, 2012). In this context, studying tourist satisfaction has profound theoretical significance: it goes beyond the traditional category of service management and becomes a core hub connecting cultural heritage value transmission, tourists’ emotional experiences, and the effectiveness of political education. It provides a key perspective for understanding the vitality of red tourism in contemporary society. Previous studies on red tourism tourist satisfaction were mostly based on classical theoretical frameworks, which are universal but failed to capture the political, educational, and emotional characteristics unique to red tourism (Zhou et al., 2025). In addition, most studies adopt a “single perspective,” focusing either on the influence of “hard environment” such as physical facilities (Xu, 2016), or discussing interpretive services in isolation (Shi, 2020), extant research has predominantly used classical, generic models such as SERVQUAL, with a primary focus on evaluating the baseline quality of services and the adequacy of tangible facilities within tourist destinations. Although this paradigm provides a reliable means of capturing universal dimensions of service performance, it remains inadequate for uncovering and interpreting the deeper and uniquely situated experiential values associated with red tourism—namely, spiritual edification, affective resonance, and political-cultural identification. Put differently, while classical frameworks may account for the surface-level determinants of whether visitors perceive services as “satisfactory,” they fall short of elucidating the underlying mechanisms through which red tourism engenders transformative experiences characterized by “education through travel” and the subtle yet profound effects of “silent influence.”

This study seeks to integrate site development effectiveness into the traditional service quality evaluation framework, systematically assessing the effectiveness of Guangzhou’s red tourism site development and identifying the key determinants of visitor satisfaction. Combining expert assessments with big-data analysis, the study quantifies the interactive mechanism between satisfaction and the perceived importance of development factors, and proposes targeted strategies for upgrading Guangzhou’s red tourism. The findings aim to provide theoretical grounding for enhancing the development effectiveness of red tourism sites in Guangzhou and, by extension, across China.

## 2 Literature review

### 2.1 Fundamental theories of red tourism development

Red tourism is a uniquely Chinese form of tourism with “red culture” as its central theme. The term “red tourism” was first introduced in Jiangxi Province in 1996. With the strong support of the Chinese government, red tourism has gradually emerged as a research hotspot in academia. A comprehensive review of relevant literature indicates that existing studies primarily focus on the connotative characteristics, constraining factors, and development strategies for red tourism in the new era (Song, 2025). Regarding the definition of red tourism, most scholars rely on the National Red Tourism Development Plan Outline issued by the Chinese government. According to this framework, red tourism is defined as a form of tourism that takes revolutionary memorial sites, monuments, and the revolutionary spirits they embody as attractions, and organizes activities for visitors to engage in sightseeing, learn revolutionary history, receive education on revolutionary traditions, become spiritually inspired, relax physically and mentally, and broaden their life experiences. For example, Hu et al. (2005) define red tourism as a type of tourism that aims to visit red tourism scenic sites, simultaneously pursuing both educational and economic benefits. Gu et al. (2007) conceptualize red tourism as being rooted in revolutionary narratives, centered on memorials and locations associated with the founding of the Chinese Communist Party, the War of Resistance against Japan, the Liberation War, and the founding of the People’s Republic of China, while generating economic, social, and ecological benefits. Li et al. (2010) drawing on the broader context of China’s economic reforms and opening-up since 1978, examined how heritage is interpreted at specific red tourism sites and analyzed how China uses red tourism to promote communist heritage, thereby reassessing the role and meaning of red tourism.

Moreover, due to its distinctive Chinese characteristics, systematic theoretical research on red tourism remains rare in the international academic community. Nonetheless, various countries have attached great importance to unique resources such as historical events and wartime culture, actively developing tourism related to cultural heritage. For instance, Caraba (2011) compared European communist heritage tourism with China’s red tourism, analyzing their origins, development trajectories, and challenges, while seeking to provide a theoretical foundation for the study of communist heritage tourism in post-communist Central and Eastern European countries. Duncan (2017) using Bucharest as a case study, examined how the heritage of communism and revolution became a focal point of interest for post-communist tourists. Similarly, Sima (2017) through a case study of Romania’s capital, Bucharest, identified and analyzed the gaps and interconnections among different stakeholders in communist heritage tourism.

### 2.2 Research on the development effectiveness of red tourism sites

Research on the development effectiveness of red tourism sites can be broadly divided into two categories: one focusing on the influencing factors of site construction, and the other emphasizing the core value concepts of red tourism. Zhang and Yu (2009) constructed an evaluation index system for the core competitiveness of red tourism, encompassing seven dimensions: government support capacity, management coordination capacity, image attractiveness, market influence, facility support capacity, product competitiveness, and cultural appeal. They used the Analytic Hierarchy Process (AHP) to measure the core competitiveness of red tourism. Zuo et al. (2017) using survey data collected from residents living near the Jinggangshan Scenic Area in China, developed two competing theoretical models integrating political trust, institutional trust, and cultural trust, in order to examine community support for the development of red tourism. Chai (2020), taking Hainan Province of China as a case, explored the locational conditions and implementation pathways for integrating red tourism with the construction of marine characteristic towns, drawing on analyses of local data. With the advancement of research, scholars have increasingly attempted to adopt the theory of value co-creation, applying big-data analysis and content analysis to systematically investigate the core values of red tourism development. For example, Chi et al. (2021), using Jinggangshan Scenic Area as a case study, constructed a framework based on historical and cultural heritage, and used multiple regression analysis to identify a value co-creation mechanism in red tourism resource development, one that fosters cognition of red history and culture, emotional connection, and value shaping. Lin and Lin (2021) argued that the fundamental logical principle of developing red tourism in the new era lies in the transmission of the “red gene,” which constitutes the theoretical core for excavating the intrinsic value of red tourism resources.

### 2.3 Research on visitor satisfaction in red tourism sites

Visitor satisfaction represents a holistic evaluation of the tourism experience and serves as a vital channel for understanding the interaction between destinations and visitors. Prior research demonstrates that satisfaction is shaped by multiple factors, and examining these determinants is essential for improving satisfaction and, in turn, evaluating the effectiveness of site development (Liu et al., 2022; Tussyadiah, 2014). As early as the 1970s, international scholarship began examining visitor satisfaction across diverse tourism destinations. For example, Pizman and Millman (1993) investigated seaside destinations, identifying eight factors influencing satisfaction, including beach quality, hospitality, accommodation facilities, and the degree of commercialization. Bowen (2001) through a systematic study, summarized six key determinants of satisfaction: expectations, attributes, fairness, visitor emotions, among others.

In the context of red tourism, Xu and Li (2021) identified five primary factors influencing visitor satisfaction: educational value, experiential quality, attractiveness, service quality, and environmental conditions. Wang and Hu (2020) observed that perceptions of red culture vary significantly across visitor types, though visitor conditions, site service development, and cultural attributes exert measurable effects. Ren and Gao (2023) differentiated between perceived satisfaction factors—such as tourism programs, infrastructure, and cultural connotation—and influencing factors, including visitor conditions, guide services, transport, and red resources. Ye (2024), through questionnaire data and IPA quadrant analysis, found relatively high visitor satisfaction at the Former Site of the Peasant Movement Institute in Guangzhou, yet with scope for further improvement, and accordingly proposed optimization measures.

This study synthesizes the core theoretical frameworks of red tourism. Red tourism development is characterized by the deep integration of political, educational, and cultural functions, evolving beyond its initial focus on patriotic education to encompass cultural transmission and regional economic coordination. In evaluating development effectiveness, existing research primarily addresses infrastructure completeness, cultural connotation, service quality, and visitor recognition of the experience. In terms of satisfaction, prior studies emphasize the combined influence of expectations, perceived quality, and value recognition. Red tourism satisfaction, however, is uniquely dependent upon emotional resonance, narrative persuasiveness, and political-cultural identification. Drawing on this literature, the present study identifies the key factors affecting both development effectiveness and visitor satisfaction, which serves as the theoretical basis for subsequent analysis.

## 3 Research design and methodology

This study incorporates visitor satisfaction within the broader framework of red tourism site development effectiveness, with the aim of constructing an evaluative model for both dimensions and advancing innovation in red tourism research paradigms. Guangzhou, as the birthplace of the modern democratic revolution and a national central city, possesses red tourism resources of high historical significance, comprehensive typological variety, and pronounced spatial concentration (Song, 2010). It is both a core node of China’s red tourism system and a representative case of integrated urban cultural–tourism development, thus offering considerable theoretical and practical value as a research subject.

The study focuses on major red tourism sites within Guangzhou, with particular attention to infrastructure, cultural presentation, and service quality as indicators of development effectiveness. From the visitor’s perspective, it empirically examines the determinants of satisfaction, without extending to regional economic spillover effects or cross-city comparative analysis.

The research process unfolds in three stages. The first stage involves expert interviews, consolidating perspectives from government, industry, and academia on key development elements, thereby establishing the foundation for quantitative analysis. The second stage comprises questionnaire surveys targeting both site managers and visitors, aimed at evaluating satisfaction and perceived importance regarding site development factors. The third stage entails statistical analysis, applying survey data to assess the effectiveness of Guangzhou’s red tourism site development and corresponding visitor satisfaction.

### 3.1 Expert interviews: exploring the guidelines for constructing red tourism sites in Guangzhou

To construct evaluation criteria for Guangzhou’s red tourism scenic areas, expert interviews were first conducted to understand how practitioners assess development effectiveness. A purposive sampling approach was adopted to recruit experts with over 10 years of practical experience in planning, management, or research on red tourism scenic areas in Guangzhou. The panel consisted of five experts: two administrative officials from the Bureau of Culture and Tourism and a municipal cultural center, one professor of tourism management, one landscape planning specialist, and one professor of ideological and political education. Table 1 presents the background information of the interviewees.

**Table 1:** Expert demographic information (*N* = 5).

ID	Gender	Years of experience	Professional background
Expert 1	Female	21	Administrative expert in the cultural and tourism sector; policy-making and tourism resource development
Expert 2	Male	17	Researcher at a municipal cultural center; event organization and brand promotion for red tourism scenic areas
Expert 3	Female	19	Professor of tourism management, teaching and research on red tourism scenic areas
Expert 4	Male	13	Planner at a landscape design institute; scenic area environment planning and exhibition design
Expert 5	Female	18	Professor of ideological and political education; research and teaching in red culture communication and education

Interviews were scheduled in advance by telephone; interview guides and a summary of preliminary literature findings were emailed to participants beforehand. Each interview lasted approximately 1 h and was conducted either in the experts’ institutional meeting rooms or via online video conference. With participants’ consent, interviews were audio-recorded for subsequent analysis.

A semi-structured interview format was used, focusing on the central question: *“How should the effectiveness of Guangzhou’s red tourism scenic area development be evaluated?”* Responses from the five experts were synthesized from the literature review, and after eliminating overlaps, 27 distinct indicators were identified (see Table 2). These indicators formed the basis of the second-stage questionnaire survey.

**Table 2:** Consolidated elements of construction effectiveness in Guangzhou’s red tourism sites.

No.	Indicator	Definition
Q1	Architectural Style Highlights Red Culture	Buildings and spatial design emphasize red cultural characteristics
Q2	Overall Coordination of Site Facilities	Public facilities are consistent with the scenic area’s overall style
Q3	Site Environment Aligns with Red Culture	The overall environment reflects the theme of red culture
Q4	Strong Red Cultural Atmosphere	Surrounding environment conveys a strong red cultural ambiance
Q5	Abundant Red Cultural Resources	Rich red cultural resources within the scenic area
Q6	Good Protection of Red Cultural Heritage	Revolutionary relics and sites are well preserved
Q7	Diverse Red Cultural Exhibitions	Multiple forms of exhibitions for red cultural resources
Q8	Activities Focus on Cultural Content	Organized activities highlight red cultural meaning
Q9	Red Cultural Features in Creative Products	Souvenirs and cultural products embody red culture
Q10	Red Cultural Content in Promotional Materials	Brochures, media, and slogans reflect red culture
Q11	Red Cultural Features in Staff Attire	Employees wear uniforms with red cultural elements
Q12	Site Operations are Well-Organized and Standardized	Business activities within the scenic area are well-regulated
Q13	Consistent Style of Operating Venues	Shops and facilities align with the scenic area’s overall style
Q14	Clean and Hygienic Site Environment	Scenic area environment is clean and sanitary
Q15	Good Safety Conditions at the Site	The area ensures visitor safety
Q16	Staff Behavior is Standardized	Staff behavior is professional and appropriate
Q17	Visitors Behave Civilly and Appropriately	Visitors respect red culture and behave appropriately
Q18	Site Services are Well-Organized	Services provided are organized and regulated
Q19	Red Cultural Study Activities are Well-Organized	Frequent and impactful educational programs are organized
Q20	Objective and Authentic Red Cultural Content	Focus on maintaining the authenticity of red cultural heritage
Q21	Systematic Red Cultural Inheritance	Emphasis on passing down red culture with clear themes
Q22	Immersive Red Cultural Atmosphere	Activities create immersive cultural experiences for visitors
Q23	Positive Staff Attitude	Staff demonstrate enthusiasm and proactive service
Q24	Extensive Red Cultural Promotion	Emphasis on promoting red culture widely
Q25	Standardized Red Cultural Interpretation	Staff deliver accurate and engaging explanations of red culture
Q26	Commemorative Activities Align with the Theme	Commemorative events align with red cultural themes
Q27	Deep Exploration of Red Cultural Content	The spirit of red culture is meaningfully highlighted

### 3.2 Survey: evaluating tourist satisfaction and importance of red tourism sites

The questionnaire comprised two sections: demographic information and evaluation items. The demographic section included gender, age, education level, political affiliation, occupation, monthly income, and place of residence. The second section was based on the 27 indicators identified in the expert interviews, with respondents rating each indicator in terms of both satisfaction and importance. A 5-point Likert scale was adopted, ranging from 1 = “very dissatisfied/unimportant” to 5 = “very satisfied/important.”

Given the focus on assessing the effectiveness and satisfaction of Guangzhou’s red tourism scenic area development, participants were required to be either experienced visitors or staff familiar with the overall conditions of these scenic areas. The survey was distributed via the “Questionnaire Star” platform on WeChat, with on-site researchers available to provide guidance. Data collection was conducted for 2 months from September to November 2024. A total of 225 questionnaires were distributed; 217 valid responses remained after excluding ineligible or incomplete submissions, yielding an effective response rate of 96.4%. The sample size exceeded a minimum threshold of 200 for human behavior research and met the requirements for factor analysis adequacy (Arrindell and Van der Ende, 1985).

Demographically, 47.5% of respondents were male and 52.5% were female. Youth and young adults constituted 50.7% of the sample. Participants with junior or senior high school education made up 30.3%. Party members or members of democratic parties constituted 28.8%. In terms of residence, 35.5% lived in urban districts and 24.9% in suburban or township areas.

### 3.3 Statistical analysis: tests of reliability, validity, and consistency of the survey data

Using SPSS, we analyzed the survey data to identify factors influencing development effectiveness and visitor satisfaction at Guangzhou’s red tourism sites. We conducted exploratory factor analysis (EFA) and importance–performance analysis (IPA) to provide decision support for improving site effectiveness and visitor satisfaction.

The data were adequate for factor analysis (Kaiser–Meyer–Olkin = 0.910). Bartlett’s test of sphericity was significant: χ^2^(351) = 6200.86, *p* < 0.001. This indicates that the measurement indicators exhibit good intercorrelations and are suitable for factor analysis (Gorsuch, 1983). The overall reliability (Cronbach’s *α*) of the 27-item scale is 0.997, and the cumulative variance explained reaches 92.089%, demonstrating very high reliability–validity and internal consistency (Bryant and Yarnold, 1995).

The study further uses IPA to examine the performance and importance of the development effectiveness of Guangzhou’s red tourism sites. Importance–performance analysis (IPA) plots the mean importance and satisfaction scores for each indicator on a two-dimensional plane: the x-axis represents performance/satisfaction, and the y-axis represents perceived importance. The grand means of satisfaction and importance constitute the central coordinates of the strategic matrix, which partitions the plane into four quadrants. Each indicator is then classified into a quadrant based on its scores, thereby informing subsequent strategy formulation and resource allocation (Azzopardi and Nash, 2013; Guadagnolo, 1985).

## 4 Research results and discussion

### 4.1 Research results

#### 4.1.1 Factor analysis and construct naming

Using principal component analysis with varimax rotation, we extracted and named the latent constructs underlying the 27 items. After rotation, three factors emerged. The eigenvalues for factors 1–3 were 9.281, 8.024, and 7.559, respectively, accounting for 34.38, 29.72, and 27.99% of the variance (cumulative variance explained = 92.09%). See Table 3 for details.

**Table 3:** Factor analysis of the development effectiveness of Guangzhou’s red tourism sites.

Visitor satisfaction construction element	Management behavioral norms	Red cultural content	Landscape facilities
Q19: Red Cultural Study Activities are Well-Organized	0.778		
Q7: Diverse Red Cultural Exhibitions	0.733		
Q18: Site Services are Well-Organized	0.721		
Q17: Visitors Behave Civilly and Appropriately	0.720		
Q25: Standardized Red Cultural Interpretation	0.688		
Q16: Staff Behavior is Standardized	0.681		
Q22: Immersive Red Cultural Atmosphere	0.676		
Q11: Red Cultural Features in Staff Attire	0.655		
Q6: Good Protection of Red Cultural Heritage	0.640		
Q5: Abundant Red Cultural Resources	0.632		
Q10: Red Cultural Content in Promotional Materials	0.628		
Q23: Positive Staff Attitude	0.624		
Q24: Extensive Red Cultural Promotion		0.560	
Q1: Architectural Style Highlights Red Culture		0.833	
Q9: Red Cultural Features in Creative Products		0.792	
Q21: Systematic Red Cultural Inheritance		0.744	
Q4: Strong Red Cultural Atmosphere		0.734	
Q20: Objective and Authentic Red Cultural Content		0.632	
Q26: Commemorative Activities Align with the Theme		0.629	
Q27: Deep Exploration of Red Cultural Content		0.598	
Q8: Activities Focus on Cultural Content		0.586	
Q15: Good Safety Conditions at the Site			0.856
Q14: Clean and Hygienic Site Environment			0.776
Q12: Site Operations are Well-Organized and Standardized			0.678
Q2: Overall Coordination of Site Facilities			0.653
Q13: Consistent Style of Operating Venues			0.627
Q3: Site Environment Aligns with Red Culture			0.611
Eigenvalue	9.281	8.024	7.559
Variance explained (%)	34.376	29.717	27.997
Cumulative variance explained (%)	92.089		
Cronbach’s α	0.997		

As presented in Table 3, factor 1 extracted 12 construction elements, including “Q19: Red Cultural Study Activities are Well-Organized,” “Q7: Diverse Red Cultural Exhibitions,” “Q18: Site Services are Well-Organized,” “Q17: Visitors Behave Civilly and Appropriately,” and “Q25: Standardized Red Cultural Interpretation.” Comprehensive analysis reveals that these elements are closely associated with behavioral norms within scenic areas. Therefore, this factor was labeled “Management Behavioral Norms.”

Factor 2 extracted nine elements, including “Q24: Extensive Red Cultural Promotion,” “Q9: Red Cultural Features in Creative Products,” “Q21: Systematic Red Cultural Inheritance,” “Q20: Objective and Authentic Red Cultural Content,” and “Q26: Commemorative Activities Align with the Theme.” These elements are consistently associated with the cultural connotations of scenic areas. Hence, this factor was labeled “Red Cultural Connotation.”

Factor 3 extracted six elements, including “Q15: Good Safety Conditions at the Site,” “Q2: Overall Coordination of Site Facilities,” “Q3: Site Environment Aligns with Red Culture,” “Q13: Consistent Style of Operating Venues,” and “Q12: Site Operations are Well-Organized and Standardized.” These elements primarily concern landscape and facility construction; therefore, this factor was labeled “Landscape and Facilities.”

#### 4.1.2 Influencing factors of visitor satisfaction

This study applies multiple regression analysis to explore the relationship between the construction elements and visitor satisfaction at Guangzhou’s red tourism sites. The relationship between the independent variables and visitor satisfaction is analyzed to determine whether multicollinearity exists, and a regression model is established. In the regression model, the dependent variable (y) is visitor satisfaction at Guangzhou’s red tourism sites, and the independent variables (xn) are x₁, “Management Behavioral Norms,” x₂, “Red Cultural Connotation,” and x₃, “Landscape and Facilities.” The test results are shown in Table 4.

**Table 4:** Analysis of the impact of basic constructs on visitor satisfaction.

Model	Unstandardized coefficients		Standardized coefficients	t	p	Collinearity statistics		R^2^
	B	Standard error	Beta			Tolerance	VIF	
(Constant)	1.530	0.138		11.112	0.000			0.503
x₂: Red Cultural Connotation	0.421	0.047	0.455	9.034	0.000	0.478	2.091	
x₁: Management Behavioral Norms	0.222	0.049	0.209	4.498	0.000	0.562	1.780	
x₃: Landscape and Facilities	0.096	0.037	0.114	2.636	0.000	0.645	1.550	

As seen in Table 4, visitor satisfaction (y) at Guangzhou’s red tourism sites is significantly related to the three basic factors—“Management Behavioral Norms” (x₁), “Red Cultural Connotation” (x₂), and “Landscape and Facilities” (x₃)—through multiple regression analysis (*p* < 0.01). The collinearity statistics show tolerance values greater than 0.1 and VIF values less than 10, indicating no multicollinearity problem (Chen and Wang, 2017). This suggests a significant linear relationship between the construction factors of “Management Behavioral Norms,” “Red Cultural Connotation,” and “Landscape and Facilities” and visitor satisfaction at Guangzhou’s red tourism sites. The adjusted R^2^ for the regression model is 0.503, which indicates a relatively high explanatory power for the model. The regression model is as follows:


(1) $$y=1.530+0.42{\mathrm{1x}}_{2}+0.222\;x_{1}+0.096\;x_{3}$$

Equation 1 shows that “Red Cultural Connotation” (x₂) has the strongest effect on tourist satisfaction (*β* = 0.421), followed by “Management Behavioral Norms” (*β* = 0.222) and “Landscape and Facilities” (*β* = 0.096).

#### 4.1.3 IPA analysis of the red tourism sites’ development effectiveness

Based on the questionnaire data, the importance and satisfaction scores and their means for the 27 evaluation indicators of visitors’ perceptions of the development effectiveness of Guangzhou’s red tourism sites are calculated. The specific values are shown in Table 5.

**Table 5:** IPA scores for the development effectiveness of red tourism sites.

No.	Indicator	Importance		Satisfaction		Strategy alignment
		Mean	Rank	Mean	Rank	
Q1	Architectural Style Highlights Red Culture	4.25	21	4.01	2	P
Q2	Overall Coordination of Site Facilities	4.26	20	4.00	3	P
Q3	Site Environment Aligns with Red Culture	4.23	24	3.94	5	P
Q4	Strong red Cultural Atmosphere	4.06	27	3.88	8	P
Q5	Abundant red Cultural Resources	4.24	23	3.83	16	L
Q6	Good Protection of Red Cultural Heritage	4.33	7	3.88	7	K
Q7	Diverse red Cultural Exhibitions	4.31	10	3.74	23	C
Q8	Activities Focus on Cultural Content	4.27	19	3.87	12	P
Q9	Red Cultural Features in Creative Products	4.29	14	3.76	20	C
Q10	Red Cultural Content in Promotional Materials	4.28	17	3.77	19	L
Q11	Red Cultural Features in Staff Attire	4.13	26	3.71	26	L
Q12	Site Operations are Well-Organized and Standardized	4.33	8	3.81	18	C
Q13	Consistent Style of Operating Venues	4.19	25	3.74	24	L
Q14	Clean and Hygienic Site Environment	4.34	5	3.91	6	K
Q15	Good safety Conditions at the Site	4.34	6	4.03	1	K
Q16	Staff Behavior is Standardized	4.31	11	3.70	27	C
Q17	Visitors Behave Civilly and Appropriately	4.24	22	3.76	21	L
Q18	Site Services are Well-Organized	4.35	3	3.88	9	K
Q19	Red cultural Study Activities are Well-Organized	4.29	15	3.71	25	C
Q20	Objective and Authentic Red Cultural Content	4.32	9	3.94	4	K
Q21	Systematic red Cultural Inheritance	4.36	1	3.88	10	K
Q22	Immersive Red Cultural Atmosphere	4.36	2	3.76	22	C
Q23	Positive Staff Attitude	4.29	16	3.83	17	C
Q24	Extensive red Cultural Promotion	4.35	4	3.83	15	C
Q25	Standardized Red Cultural Interpretation	4.31	12	3.84	14	C
Q26	Commemorative Activities Align with the Theme	4.28	18	3.88	11	P
Q27	Deep Exploration of Red Cultural Content	4.29	13	3.86	13	K
Average		4.28		3.84		

K (continue to maintain), C (priority for improvement), L (low improvement priority), and P (over-quality).

For IPA, the intersection of the x- and y-axes is set at the grand means of satisfaction and importance across the 27 indicators. Following the expectation–disconfirmation theory, the plot is divided into four quadrants that indicate different evaluative states and inform priority setting for managerial interventions.

Quadrant interpretations are as follows:

- Quadrant I (high importance, high performance): maintain.
- Quadrant II (high importance, low performance): priority for improvement.
- Quadrant III (low importance, low performance): low priority.
- Quadrant IV (low importance, high performance): potential over-investment.

**Figure 1:**
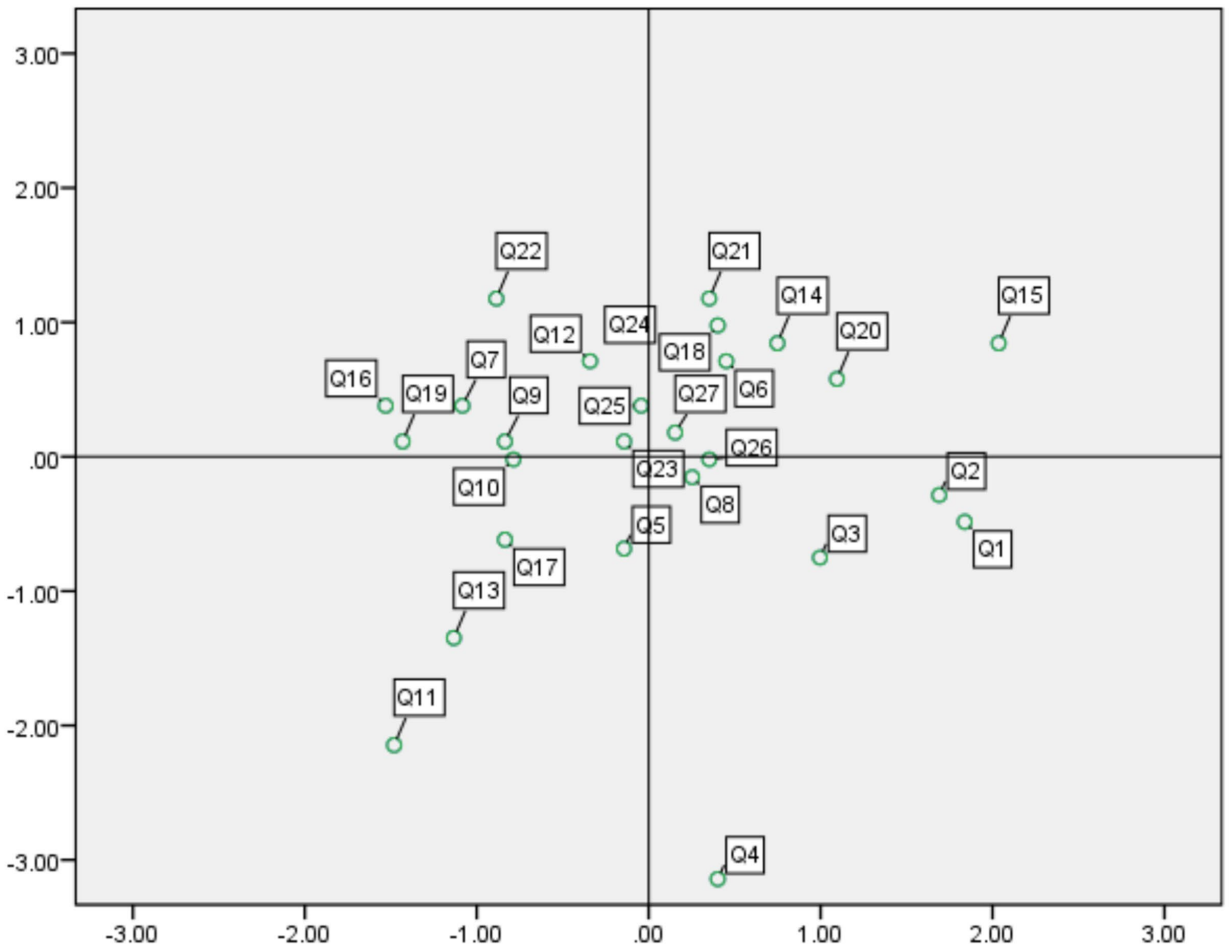
IPA strategy matrix for Guangzhou red tourism scenic area construction elements.

Indicators in the second quadrant are considered very important yet exhibit unsatisfactory performance; they require priority improvement. Indicators in the third quadrant are not recognized as particularly important, and their performance is likewise evaluated as low; they may be assigned a low improvement priority. Indicators in the fourth quadrant indicate over-quality: performance meets visitors’ expected standards, yet the indicators are not regarded as important. The IPA analysis matrix for the development effectiveness of Guangzhou’s red tourism sites is shown in Figure 1. In accordance with the IPA analysis plot, the enhancement strategies for the factors affecting the development effectiveness and satisfaction of Guangzhou’s red tourism sites are presented in Table 5.

## 5 Discussion

### 5.1 Discussion on the dimensions of scenic area construction effectiveness

This study identifies a three-dimensional evaluation system for construction effectiveness and visitor satisfaction at Guangzhou’s red tourism sites, comprising 27 indicators. The three dimensions are identified as “Management Behavioral Norms,” “Red Cultural Connotations,” and “Landscape and Facilities.” Literature review confirms that prior scholarship has repeatedly emphasized the positive role of behavioral norms in enhancing the construction effectiveness of red tourism scenic areas: standardized services ensure efficient and orderly reception processes, thereby effectively improving the visitor experience (Zhu and Song, 2023); guiding visitors to behave in a civilized and respectful manner helps maintain a solemn atmosphere and further highlights the educational function of red tourism (Tang, 2023); strict adherence to interpretive norms of red culture guarantees historical authenticity, ideological seriousness, and communicative effectiveness (Zhang and Ran, 2024). Dai and Ma (2021) argue that extensive and in-depth promotion of cultural connotations can effectively expand the social influence of scenic areas and foster a sound educational atmosphere. Shi (2020) highlights that maintaining orderly transmission and ensuring objectivity and factual accuracy are the foundations for upholding the seriousness and authority of red culture, thereby safeguarding the credibility of educational content. Wu and Li (2021) posit that the development of culturally distinctive and meaningful red cultural and creative products can extend cultural experiences, strengthen visitor memory and emotional resonance, and that organizing commemorative activities closely aligned with central themes can reinforce ritual and appeal, embedding the red spirit in people’s hearts. Similarly, Chen et al. (2025) contend that well-designed landscape and facility construction is a critical guarantee for improving the quality and efficiency of red tourism scenic areas; sound safety conditions constitute the operational foundation, overall facility coordination enhances the touring experience, and an environment that resonates with the red theme strengthens immersive educational engagement.

### 5.2 Discussion on the determinants of visitor satisfaction

Among the multiple factors influencing visitor satisfaction in Guangzhou’s red tourism scenic areas, the dimension of “Red Cultural Connotations” exerts the greatest impact, with a direct effect of 0.455. This suggests that “Red Cultural Connotations” constitute the core attraction driving visitor satisfaction in Guangzhou’s red tourism. Empirical evidence indicates that visitors not only pay attention to the authenticity of history but also value its modern interpretation and the depth of its dissemination; the authenticity and narrative depth of red culture directly determine visitors’ cognitive gains and educational experiences. The higher the authenticity and the deeper the connotations, the better visitors’ learning expectations are fulfilled (Lei and Yang, 2021). Management behavioral norms serve as the service guarantee of high-quality experiences. Covering multiple dimensions of scenic area management, Xu et al. (2016) emphasize that scientifically standardized management and services can effectively guide visitors to achieve knowledge acquisition, heightened awareness, and emotional enrichment, thereby stimulating behavioral intentions to inherit red revolutionary traditions.

### 5.3 Discussion on strategies for enhancing importance and satisfaction

As shown in Table 5, the mean score of importance across all 27 indicators is 4.28, while the mean satisfaction score is 3.84. The mean value of importance is consistently higher than that of satisfaction, reflecting that visitors hold high expectations for the construction effectiveness of red tourism scenic areas in Guangzhou, yet actual performance has not fully met such expectations. This finding also corroborates the perspectives of Wu et al. (2023) and others, who argue that while visitors maintain elevated expectations for Guangzhou’s red tourism scenic areas, multiple performance gaps persist. Ye (2024) further suggests that visitors perceive significant discrepancies between expectations and performance in terms of the depth of cultural connotation excavation and its modern expression, the normativity and professionalism of services, and the coordination of landscape facilities. Results derived from the IPA analysis indicate that indicators requiring prioritized improvement are concentrated in the domains of red cultural connotation construction and standardized service management. Field survey comparisons further reveal that instances of integrating red cultural elements into supporting facilities remain limited and lack innovation, whereas most visitors demand higher standards for the presentation forms of red resources, red elements, and red culture, ultimately resulting in relatively low satisfaction levels.

The study develops and empirically tests a three-dimensional model, “Red Cultural Connotation—Management Behavioral Norms—Landscape and Facilities,” that specifies the mechanism by which red tourism produces transformative experiences. Our results show that “Red Cultural Connotation” is the critical variable triggering deep cognitive reflection and emotional resonance (the core of transformative experience), going beyond the traditional service-quality emphasis on functional attributes. This advances knowledge by moving the study of transformative experiences from broad motivational theorizing to measurable, actionable elements of site design and management. Specifically, it clarifies that through systematic design choices—such as deepening narrative interpretation, standardizing service practices, and deliberately shaping atmospheric cues—managers can consciously guide visitors from mere “touring” toward “value identification and inner transformation.” This provides a new theoretical pathway for understanding how tourism, in the context of red tourism, can promote individual wellbeing.

## 6 Research conclusion and recommendations

### 6.1 Research conclusion

This study systematically constructed an evaluation system for the construction effectiveness of red tourism scenic areas in Guangzhou through literature research, expert interviews, and questionnaire surveys, and empirically tested its influence mechanism on visitor satisfaction. Based on comprehensive statistical analyses and expert judgment, three core findings emerge: (1) The construction effectiveness of Guangzhou’s red tourism scenic areas constitutes a multidimensional and integrated system composed of three critical dimensions—“Red Cultural Connotations,” “Management Behavioral Norms,” and “Landscape and Facilities”—together with 27 specific measurement indicators. (2) The three dimensions exert significantly differentiated impacts on visitor satisfaction. Empirically, red cultural connotations exert the strongest influence on visitor satisfaction, followed by management behavioral norms; landscape and facilities have a smaller but still significant effect. (3) Visitors generally maintain high expectations for the construction effectiveness of Guangzhou’s red tourism scenic areas, yet their perceived satisfaction has not fully met these expectations, resulting in a pronounced “expectation–perception” negative gap.

Based on these findings, the study reaches the following conclusions:

(1) Theoretically, our results demonstrate that construction effectiveness in red tourism is multidimensional rather than reducible to hardware or landscape alone. Through factor analysis, we identify three key dimensions—red cultural connotations (e.g., narrative depth, ideological expression, and exhibition quality); management behavioral norms (e.g., service professionalism, operational orderliness, and visitor civility); and landscape and facilities (e.g., environmental coordination, facility completeness, and ecological aesthetics). Together, these dimensions form a comprehensive framework for evaluating construction effectiveness in red tourism scenic areas. This model highlights that enhancing red tourism scenic areas requires not only solemn and well-maintained landscapes but also efficient behavioral norms, with cultural connotations serving as the true core competitiveness capable of resonating with visitors. This provides academia with a valuable theoretical model for understanding and assessing the overall quality of red tourism scenic areas.
(2) Empirically, this study demonstrates the differentiated paths through which each dimension influences visitor satisfaction, clarifying that “Red Cultural Connotations” are the most critical factor driving satisfaction and value recognition. Multiple regression analysis reveals that the standardized path coefficient of “Red Cultural Connotations” significantly surpasses those of the other two dimensions. This finding holds notable theoretical significance: it strongly validates the applicability of service-dominant logic in the red tourism domain—what visitors ultimately pay for and find satisfying is not merely grand architecture or beautiful environments, but rather the profound cultural value experiences and emotional resonance that scenic areas deliver. “Management Behavioral Norms” act as indispensable guarantees for cultural value delivery, while “Landscape and Facilities” function as foundational carriers, their effectiveness contingent upon robust cultural and normative underpinnings. This outcome clarifies the priority sequence of resource allocation within scenic area management, offering critical theoretical guidance for decision-making.
(3) The identification of a “high expectation–low perception” performance gap pinpoints the central challenge in the current development of Guangzhou’s red tourism scenic areas and highlights the key direction for future quality upgrading. Survey data indicate that visitors hold high reverence and learning expectations for Guangzhou’s profound revolutionary history as one of the birthplaces of the modern Chinese revolution; however, deficiencies in the depth of cultural interpretation, innovativeness of presentation, and refinement of service experiences hinder satisfaction. Consequently, future construction priorities must shift away from scale expansion and hardware upgrades toward the in-depth excavation, innovative expression, and effective dissemination of cultural connotations.

### 6.2 Research limitations and recommendations

This study provides a validated evaluation system for Guangzhou’s red tourism sites; however, several limitations should be noted. First, data were collected between September and November, and seasonal variation in site landscapes may affect visitor perceptions. Second, the sample was concentrated in Guangzhou’s core urban scenic areas; future research should test the model’s generalizability across other cities in the Greater Bay Area. Third, the evaluation system was constructed primarily through expert interviews and questionnaire surveys; subsequent studies should integrate spatial points of interest and diverse Internet-based data sources to enhance the overall reliability and validity of the research.

## Data Availability

The original contributions presented in the study are included in the article/supplementary material, further inquiries can be directed to the corresponding author.
